# Prognosis of resectable colorectal liver metastases after surgery associated with pathological features of primary tumor

**DOI:** 10.3389/fonc.2023.1181522

**Published:** 2023-05-25

**Authors:** Dawei Chen, Qingshan Li, Haibo Yu

**Affiliations:** ^1^Department of Hepatobiliary Surgery, People’s Hospital of Zhengzhou University, Zhengzhou, China; ^2^Department of Hepatobiliary Surgery, Henan Provincial People’s Hospital, Zhengzhou, China

**Keywords:** MMR, ki67, lymphovascular invasion, prognosis, nomogram

## Abstract

**Background:**

Surgery is an important means for patients with colorectal liver metastases (CRLM) to improve their long-term survival, and accurate screening of high-risk factors is crucial to guiding postoperative monitoring and treatment. With this in mind, the aim of this study was to investigate the expression levels and prognostic roles of Mismatch Repair (MMR), Ki67, and Lymphovascular invasion(LVI) in the tumor tissues of colorectal of CRLM.

**Methods:**

85 Patients with CRLM who received surgical treatment for liver metastases after colorectal cancer resection from June, 2017 and Jan, 2020 were included in this study. Independent risk factors affecting the survival of patients with CRLM were investigated using a Cox regression model and the Kaplan-Meier method, and a nomogram for predicting the OS of patients with CRLM was established according to a Cox multivariate regression model. Calibration plots and Kaplan-Meier curves were used to assess the performance of the nomogram.

**Results:**

The median survival time was 39 months (95% CI: 32.05-45.950), and MMR, Ki67 and LVI were significantly correlated with prognosis. Univariate analysis indicated that larger metastasis size (p=0.028), more than one liver metastases (p=0.001),higher serum CA199 (p<0.001), N1-2 stage (p<0.001), the presence of LVI (p=0.001), higher Ki67 (p<0.001), and pMMR predicted worse OS. In addition, synchronous liver metastasis (p = 0.008), larger metastasis size (p=0.02), more than one liver metastases (p<0.001),higher serum CA199 (p<0.001), the presence of LVI (p=0.001), nerve invasion (p=0.042) higher Ki67 (p=0.014), and pMMR (p=0.038) were each associated with worse DFS. Multivariate analysis indicated that higher serum CA199 (HR = 2.275, 95%CI: 1.302-3.975 p=0.004), N1-2 stage(HR = 2.232, 95%CI: 1.239-4.020 p=0.008), the presence of LVI (HR = 1.793, 95%CI: 1.030-3.121 p=0.039), higher Ki67 (HR = 2.700, 95%CI: 1.388-5.253\ p=0.003), and pMMR (HR = 2.213, 95%CI: 1.181-4.993 p=0.046) all predicted worse OS. Finally, synchronous liver metastasis (HR = 2.059, 95%CI: 1.087-3.901 p=0.027), more than one liver metastases ((HR =2.025, 95%CI: 1.120-3.662 p=0.020),higher serum CA199 (HR =2.914, 95%CI: 1.497-5.674 p=0.002), present LVI (HR = 2.055, 95%CI: 1.183-4.299 p=0.001), higher Ki67 (HR = 3.190, 95%CI: 1.648-6.175 p=0.001) and pMMR(HR = 1.676, 95%CI: 1.772-3.637 p=0.047) predicted worse DFS, and the nomogram achieved an effective level of predictive ability.

**Conclusion:**

This study showed that MMR, Ki67, and Lymphovascular invasion were independent risk factors for the postoperative survival of CRLM patients, and a nomogram model was constructed to predict the OS of these patients after liver metastasis surgery. These results can help surgeons and patients to develop more accurate and individualized follow-up strategies and treatment plans after this surgery.

## 1 Introduction

The liver is the main target organ for hematogenous metastasis of colorectal cancer (CRC), which is the most common malignant tumor of the digestive tract. According to Global Cancer Epidemiology Statistics published by the International Agency for Research on Cancer (IACR), colorectal cancer has become the second leading cause of death among all malignant tumors, with 935,200 deaths estimated in 2020 (1), and colorectal liver metastases (CRLM) is one of the main causes of death in patients with CRC (2). The median survival time of patients with liver metastases without surgical resection is only 6.9 months, with a 5-year survival rate of less than 5% (3), but the median survival time of CRLM patients with liver metastases who undergo surgical resection is 35 months, with a 5-year survival in the range of 30%-57% (4–7). Thus, colorectal liver metastasis (CRLM) is of critical importance in the treatment of CRC (8, 9). The object of this study is to find new risk factors of CRLM, and to establish an effective model for predicting the prognosis of initially resectable CRLM.

## 2 Materials and methods

### 2.1 Patients

**Figure 1:**
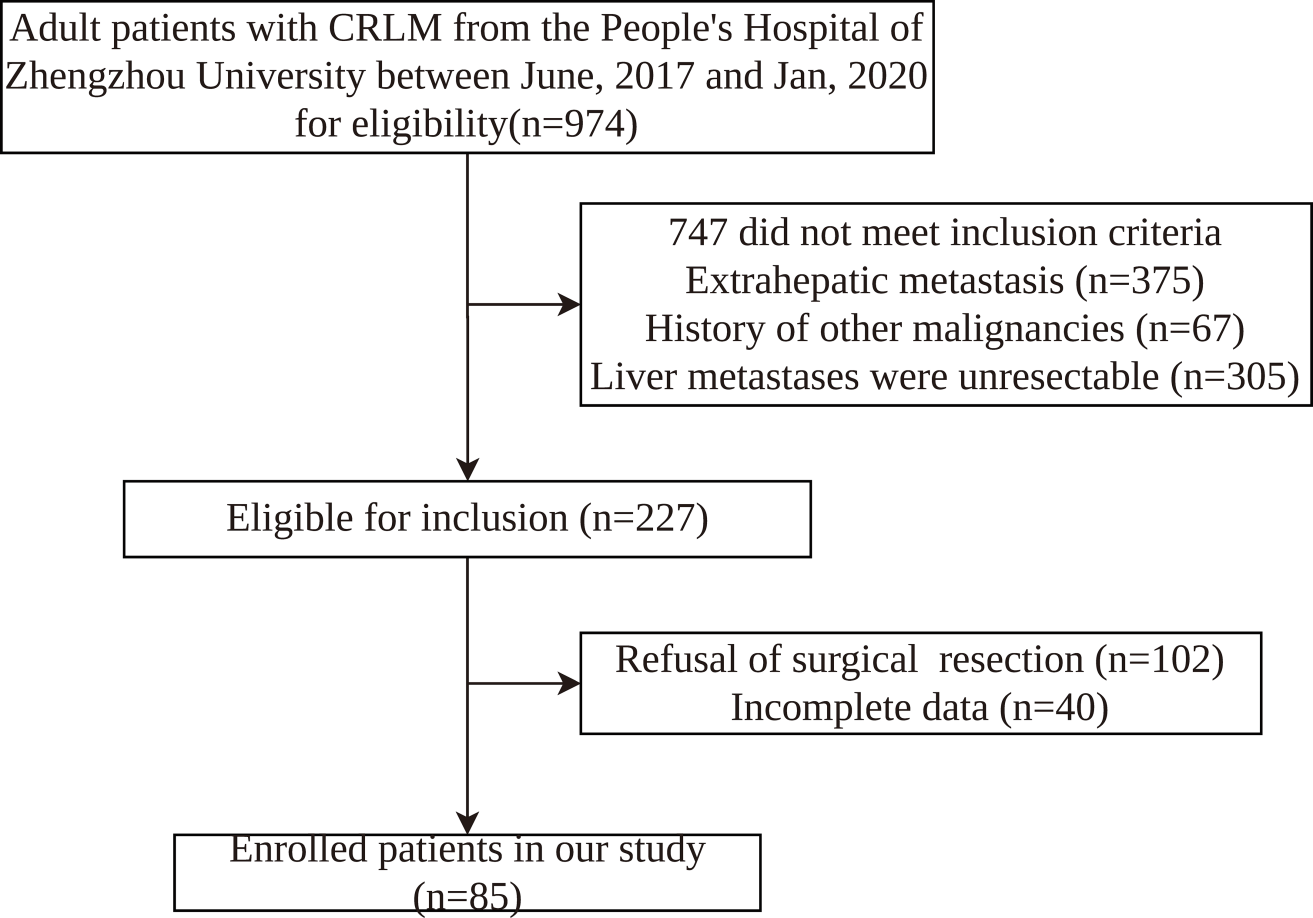
Flow chart of patient enrollment.

We retrospectively assessed adult patients from the People’s Hospital of Zhengzhou University between June, 2017 and Jan, 2020. This retrospective study was approved by the Ethics Committee of the People’s Hospital of Zhengzhou University. The inclusion criteria were as follows: (1) diagnosis of CRLM by ultrasound, CT, magnetic resonance imaging (MRI), or colorectal endoscopy; (2) primary lesion of colorectal cancer having undergone radical resection; (3) age ≥ 18 years old; (4) no history of other tumors; (5) no treatment received before admission. The exclusion criteria were: (1) receipt of emergency surgical treatment; (2) no complete clinicopathological information; (3) extrahepatic metastasis. Finally, 85 patients were enrolled in this study (**Figure 1**). The criteria for resectable CRLM were defined as follows: (1) the primary lesion having been resected with curative intent; (2) the function of remnant liver volume after hepatectomy meeting the needs of patients(The CRLM can be completely resected, two adjacent liver segments can be spared, adequate vascular inflow and outflow and biliary drainage can be preserved, and the volume of the future liver remnant will be adequate); (3) the patient having been able to tolerate hepatectomy.

The clinical characteristics of CRLM patients were collected, including age (years), sex, viral hepatitis status, timing of liver metastasis, primary lesion site, size of primary tumor (mm), size of metastases (mm), number of liver metastases, sCEA(ng/ml), sCA-199(ng/ml), N stage and T stage, degree of differentiation, tumor type, the presence of LVI (LVI), the presence of nerve invasion, ki67(%), MMR, chemotherapy regimen (5-Fu-based or Capecitabine -based).

### 2.2 Treatment and follow-up

All patients had surgical resection of liver metastases and continued to receive systemic adjuvant chemotherapy postoperatively, and all patients also received 5-FU-based or capecitabine-based systemic chemotherapy regimens such as XELOX, FOLFOX, and FOLFIRI after surgery. Surveillance for recurrence was performed every 3 to 6 months for 5 years after surgery and annually thereafter. Serum tumor markers and CT scans of the chest and abdomen were included. Local tumor recurrence was defined as the appearance of a new lesion at the margin after hepatectomy, and distant recurrence was defined as metastasis to other organs. In addition to medical records, imaging data were reviewed to determine patterns of recurrence.

### 2.3 Outcome definitions

The primary outcome event was overall survival (OS), defined as the interval from the day of liver surgery to the time of the last visit or death from any cause. Another important outcome event was disease-free survival (DFS), which was defined as the time from the day of surgery to recurrence or death, whichever occurred first.

### 2.4 Immunohistochemistry

**Figure 2:**
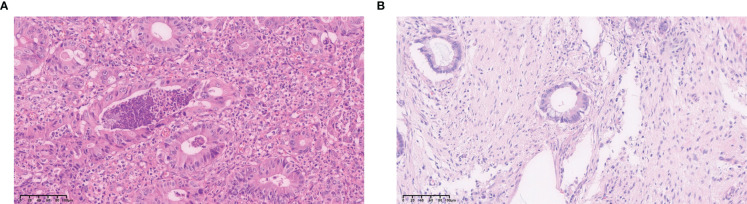
H&E staining results for CRLM primary tumor tissues. **(A)** LVI present. **(B)** LVI absent.

Pathological sections of the primary lesions of CRLM were routinely stained with H&E and immunohistochemical staining. Pathological examinations were performed by two pathologists individually, and different conclusions were resolved by discussion with a third expert. Interpretation of LVI went thusly: when cancer cells entered the tumor or the vascular or lymphatic endothelium outside the tumor as detected under a light microscope, they were diagnosed as positive for vascular tumor thrombus (**Figure 2**).

**Figure 3:**
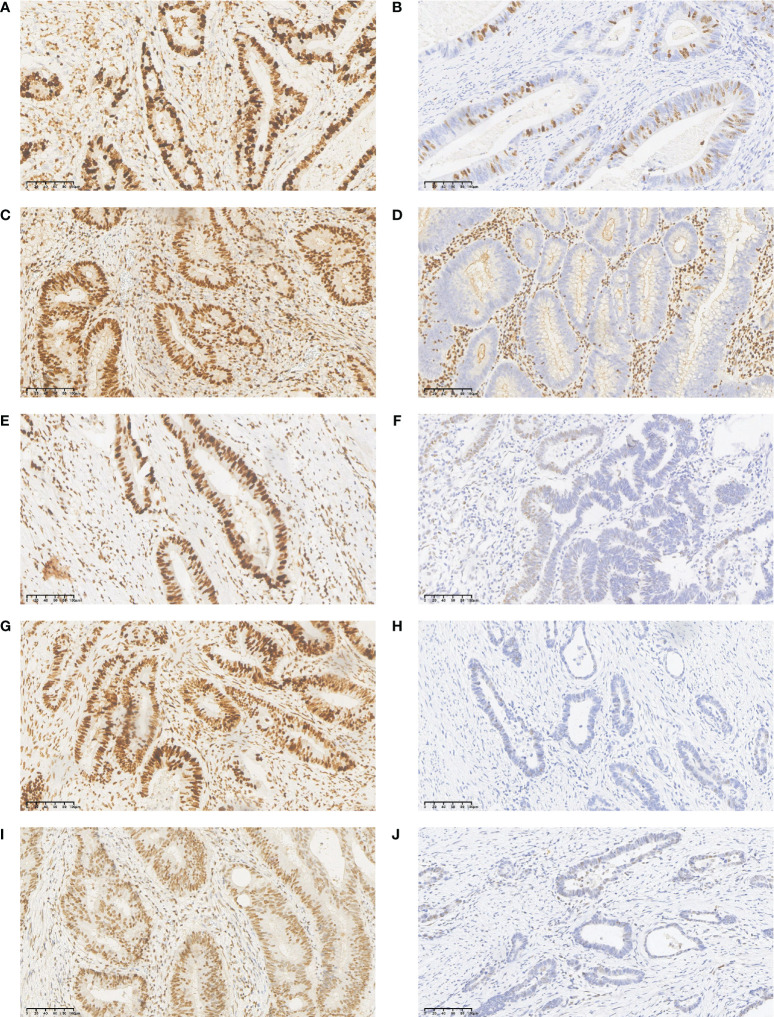
Immunohistochemistry (IHC) staining results for Ki67, MLH1, MSH2, MSH6, and PMS2 in CRLM primary tumor tissues. **(A)** high expression of Ki67; **(B)** low expression of Ki67; **(C)** normal expression of MLH1 protein; **(D)** lack of MLH1 protein expression; **(E)** normal expression of MSH2 protein; **(F)** lack of MSH2 protein expression; **(G)** normal expression of MSH6 protein; **(H)** lack of MSH6 protein expression; **(I)** normal expression of PMS2 protein; **(J)** lack of PMS2 protein expression.

Ki67-positive cells were defined as those with pale yellow to brown-yellow nuclei, as examined under a light microscope at 200x. Ten visual fields were randomly selected, and 500 tumor cells were counted per visual field. The percentage (%) of ki67 positive cells in the total number of tumor cells in these visual fields was used as the ki67 proliferation fraction (**Figures 3A, B**).

The detection of MMR status was performed primarily by immunohistochemistry to detect the expressions of four key proteins: MLH1, MSH2, MSH6, and PMS2 in the surgically removed primary tumor tissue. Normal colorectal epithelium was used as a positive control, and phosphate buffer (PBS) was used as a negative control. The results showed that the MMR protein was localized in the nucleus, and positive cells were defined as brown or yellow-brown granules in the nucleus. Five fields were randomly selected under the microscope at 200× magnification, and 100 cells were counted in each field to calculate the average proportion of positive cells. Scores were assigned according to the proportion of positive cells and the intensity of cell staining as follows. For staining intensity, we recorded 0 points for no staining, 1 point for light yellow, 2 points for brown, and 3 points for tan. For the proportion of positive cells, no positive cells received 0 points, ≤ 10% 1 point; 10% -50% 2 points; 50% -75% 3 points; and >75% 4 points. A product of two scores > 3 indicated normal expression of MMR protein, and ≤3 indicated lack of MMR protein expression (**Figures 3C–J**). A Mismatch Repair proficiency (pMMR) score was defined as the expression of MLH1, MSH2, MSH6 and PMS2, and the absence of at least one of these proteins was defined as Mismatch Repair Deficiency (dMMR).

### 2.5 Statistical analysis

Prior to all analysis categorical variables were expressed as frequencies, and continuous variables were expressed as mean ± standard deviation (SD). The surv_cutpoint() function of the survminer package in R software was used to calculate the best cut-off value for ki67. A Cox proportional hazards regression model was then used for univariate and multivariate analysis of clinicopathological factors, and a nomogram was constructed from the Cox multivariate analysis. The predictive results of the nomogram then were evaluated using a calibration curve. All statistical analysis was performed using SPSS 25.0 (SPSS, Inc., Chicago, IL, USA) and R version 4.2.1 (http://www.r-project.org).

## 3 Results

### 3.1 Clinical characteristics

Among the 85 participants, 64.7% were male, and the median patient age was 57 (range 22-80) years. The primary tumor was located in the left colon in 64 patients (75.3%) and in the right in 21 (24.7%) patients. Liver metastases were confirmed in 32 patients (37.6%) at the same time that colorectal cancer was identified, with an average metastasis diameter of 32.12 ± 16.433. The postoperative 1-year OS rate was 88.2%, the postoperative 3-year OS rate was 57.6%, and the postoperative 1-year OS rate was 32.7%. Finally, the median OS was 39 ± 3.5 months after follow-up.

The relationship between the expression of Ki67, MMR and LVI in the primary tumor tissues of CRLM patients and clinicopathologic features: 26 patients had high Ki67 expression and 59 patients had low Ki67 expression; There were 20 patients with dMMR status and 65 patients with pMMR status. LVI was positive in 39 patients and negative in 46 patients. The expression of Ki67 in the Primary tumor tissues of patients is related to the primary lesion site, size of primary tumor and MMR. The expression of MMR was associated with N stage, LVI, and Ki67.LVI expression is associated with Nerve invasion and MMR status(**Tables S1**-**S3**).

### 3.2 Optimal cutoff value of Ki67

**Figure 4:**
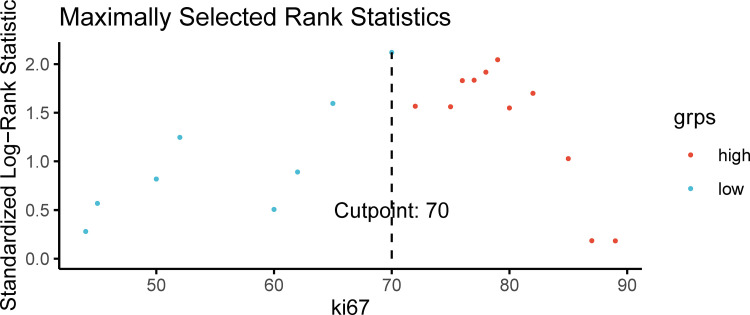
The optimal Ki67 cutoff value was identified to be 70%.

A proliferation index of Ki67 of 70% was determined to be the optimal cutoff value according to the correlation between the proliferation index of Ki67 and OS (**Figure 4**). The patients with CRLM were thus divided into high-Ki67(≥70%) and low-Ki67 (<70%) groups.

### 3.3 Univariate and multivariate analysis of OS

Univariate analysis indicated that larger metastasis size (p=0.028), more than one liver metastases (p=0.001),higher serum CA199 (p<0.001), N1-2 stage (p<0.001), the presence of LVI (p=0.001), higher Ki67 (p<0.001), and pMMR predicted worse OS. Multivariate analysis showed that higher serum CA199 (HR = 2.2752.389, 95%CI: 1.302-3.9751.378-4.143 p=0.0040.003), N1-2 stage(HR = 2.2322.204, 95%CI: 1.239-4.0201.228-3.954 p=0.008), the presence of LVI (HR = 1.7931.955, 95%CI: 1.030-3.1211.136-3.362 p=0.0390.015), higher Ki67 (HR = 2.7002.931, 95%CI: 1.388-5.253\1.521-5.647 p=0.0030.001), and pMMR (HR = 2.2132.328, 95%CI: 1.181-4.9931.042-5.201 p=0.0460.039) were independent risk factors for OS (**Table 1**).

**Table 1:** Univariate and multivariate analysis of risk factors in relation to OS in CRLM.

Parameter		N	Univariate analysis		Multivariate analysis	
			HR(95%CI)	p value	HR(95%CI)	*P* value
**Sex**	Female	30	1.099(0.681-1.771)	0.700		
	Male	55	Ref			
**Age(years)**	<60	45	Ref			
	≥60	40	1.347(0.856-2.120)	0.198		
**Viral hepatitis**	positive	9	1.390(0.664-2.908)	0.383		
	negative	76	Ref			
**Timing of liver metastases**	Synchronous liver metastases	53	Ref			
	Metachronous liver metastases	32	0.674(0.419-1.084)	0.104		
**Primary lesion site**	Left hemi-colon	63	Ref			
	Right hemi-colon	22	1.584(0.961-2.610)	0.071		
**Size of primary tumor (mm)**	<50	53	Ref			
	≥50	32	1.564(0.987-2.480)	0.057		
**Size of metastases (mm)**	<30	48	Ref			
	≥30	37	**1.670**(**1.057-2.636**)	*0.028	1.475(0.865-2.514)	0.153
**number of liver metastases**	1	52				
	>1	33	**2.326(1.426-3.792)**	*0.001	1.522(0.911-2.542)	0.109
**sCEA(ng/ml)**	<5	18	Ref			
	≥5	67	1.456(0.813-2.607)	0.206		
**sCA-199(ng/ml)**	<35	51	Ref			
	≥35	34	**2.472**(**1,537-3.975**)	*< **0.001**	**2.275**(1.**302-3.975**)	*0.004
**N stage**	N0	31	Ref			
	N1-2	54	**3.007**(**1.722-5.250**)	*< **0.001**	**2.232**(**1.239-4.020**)	*0.008
**T stage**	T1-2	7	Ref			
	T3-4	78	1.637(0.594-4.508)	0.559		
**Degree of differentiation**	High or Moderately differentiation	77	Ref			
	Poorly differentiation	8	1.464(0.667-3.214)	0.342		
**Tumor types**	Uplift type	7	1.331(0.862-2.056)	0.197		
	Ulcer type	74				
	invasive	4	Ref			
**Lymphovascular invasion**	Absent	40	Ref			
	Present	45	**2.355**(**1.443-3.842**)	*0.001	**1.793**(**1.030-3.121**)	*0.039
**Nerve invasion**	Negative	36	Ref			
	Positive	49	1.558(0.964-2.517)	0.070		
**Ki67(%)**	<70%	51	Ref			
	≥70%	34	**3.200**(**1.738-5.890**)	*< **0.001**	**2.700**(**1.388-5.253**)	*0.003
**MMR**	dMMR	16	Ref			
	pMMR	69	**4.899**(**2.378-10.092**)	*< **0.001**	**2.213**(**1.181-4.993**)	*0.046
**Chemotherapy regimen**	5-Fu-based	32	Ref			
	Capecitabine -based	53	1.560(0.960-2.535)	0.073		

* Statistically significant correlation. sCEA, preoperative serum CEA; sAFP, preoperative serum CA199; pMMR, Mismatch Repair Proficiency; dMMR, Mismatch Repair Deficiency; Ref, reference variables. Bold values means statistical differences.

**Figure 5:**
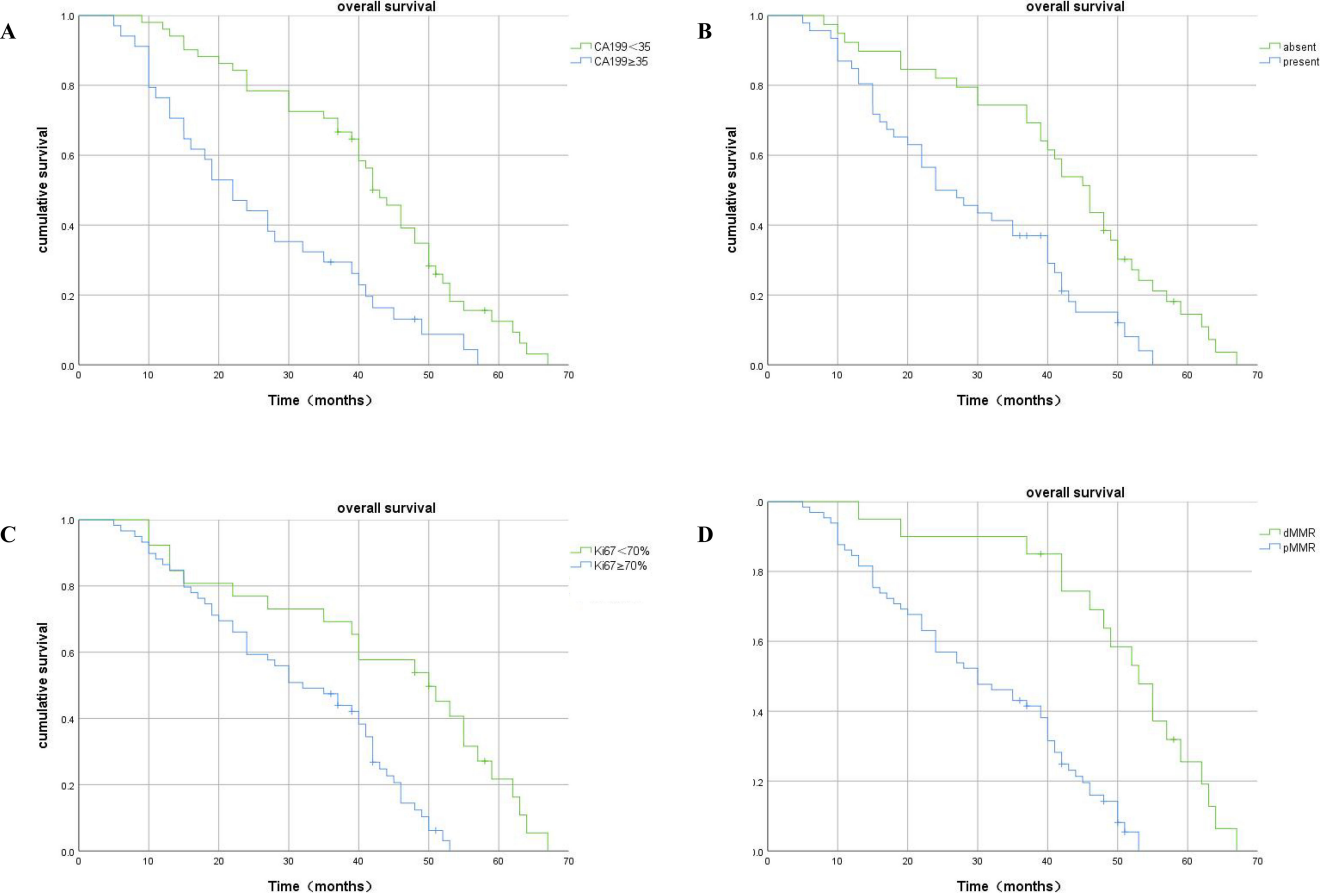
Kaplan–Meier plots of OS contrasting high versus low expression of CA199, absence versus presence of LVI, high-Ki67 versus low-Ki67, and dMMR versus pMMR. **(A)** Association between OS and serum CA199 expression: patients with high serum CA199 expression had significantly shorter OS (p<0.001). **(B)** Association between OS and LVI expression: patients with LVI had significantly shorter OS (p<0.001). **(C)** Association between OS and Ki67 expression: patients with higher Ki67 metastasis expression had significantly shorter OS (p<0.001). **(D)** Association between OS and MMR expression: patients with pMMR had significantly shorter OS (p<0.001).

Kaplan–Meier analysis showed that patients with a high level of serum CA199, high expression of Ki67, the presence of LVI, and pMMR had significantly shorter OS duration than CRLM patients with low serum CA199, low expression of Ki67, the absence of LVI, and dMMR (**Figure 5**).

### 3.4 Univariate and multivariate analysis of DFS

Univariate analysis showed that synchronous liver metastasis (p = 0.010.008), larger metastasis size (p=0.02), more than one liver metastases (p<0.001),higher serum CA199 (p<0.001), the presence of LVI (p=0.001), nerve invasion (p=0.042) higher Ki67 (p=0.014), and pMMR (p=0.038) were associated with worse DFS (**Table 2**). synchronous liver metastasis (HR = 2.0590.475, 95%CI: 1.087-3.9010.258-0.876 p=0.0270.017), more than one liver metastases (HR =2.025, 95%CI: 1.120-3.662 p=0.020),higher serum CA199 (HR =2.9143.034, 95%CI: 1.497-5.674 1.607-5.730 p=0.0020.01), present LVI (HR = 2.0552.796, 95%CI: 1.183-4.2991.399-5.585 p=0.0010.004) and, higher Ki67 (HR = 3.1903.099, 95%CI: 1.648-6.1751.610-5.963 p=0.0010.001) and pMMR(HR = 1.676, 95%CI: 1.772-3.637 p=0.047)were independent risk factors for DFS in the multivariate analysis (**Table 2**).

**Table 2:** Univariate and multivariate analysis of risk factors in relation to DFS in CRLM.

Parameter		N	Univariate analysis		Multivariate analysis	
			HR(95%CI)	p value	HR(95%CI)	*p* value
**Sex**	Female	30	1.597(0.950-2.685)	0.077		
	Male	55	Ref			
**Age(years)**	<60	45	Ref			
	≥60	40	1.344(0.822-2.195)	0.238		
**Viral hepatitis**	positive	9	1.119(0.384-2.080)	0.795		
	negative	76	Ref			
**Timing of liver metastases**	Synchronous liver metastases	53	**2.096(1.210-3.631)**	*0.008	**2.059(1.087-3.901)**	*0.027
	Metachronous liver metastases	32	Ref			
**Primary lesion site**	Left hemi-colon	63	0.961(0.551-1.675)	0.888		
	Right hemi-colon	22	Ref			
**Size of primary tumor (mm)**	<50	53	Ref			
	≥50	32	1.359(0.826-2.235)	0.227		
**Size of metastases (mm)**	<30	48	Ref			
	≥30	37	**1.800**(**1.099-2.947**)	*0.020	1.441(0.814-2.553)	0.210
**number of liver metastases**	1	52	**Ref**			
	>1	33	**2.595(1.544-4.360)**	*<0.001	**2.025(1.120-3.662)**	*0.020
**sCEA(ng/ml)**	<5	18	Ref			
	≥5	67	1.371(0.753-2.497)	0.302		
**sCA-199(ng/ml)**	<35	51	Ref			
	≥35	34	**3.234**(**1.914-5.465**)	*< **0.001**	**2.914**(**1.497-5.674**)	*0.002
**N stage**	N0	31	Ref			
	N1-2	54	1.426(0.848-2.395)	0.180		
**T stage**	T1-2	7	Ref			
	T3-4	78	1.357(0.534-3.450)	0.521		
**Degree of differentiation**	High or Moderately differentiation	77	Ref			
	Poorly differentiation	8	1.590(0.722-3.502)	0.250		
**Tumor types**	Uplift type	7	1.477(0.908-2.404)	0.116		
	Ulcer type	74				
	invasive	4	Ref			
**Lymphovascular invasion**	Absent	40	Ref			
	Present	45	**2.610**(**1.519-4.485**)	*0.001	**2.055**(**1.183-4.299**)	*0.001
**Nerve invasion**	Negative	36	Ref			
	Positive	49	**1.752**(**1.021-3.006**)	*0.042	1.313(0.729-2.363)	0.365
**Ki67(%)**	<70%	51	Ref			
	≥70%	34	**2.053**(**1.160-3.635**)	*0.014	**3.190**(**1.648-6.175**)	*0.001
**MMR**	dMMR	16	Ref			
	pMMR	69	**1.864**(**1.034-3.360**)	*0.038	**1.676(1.772-3.637)**	*0.047
**Chemotherapy regimen**	5-Fu-based	32	Ref			
	Capecitabine -based	53	1.335(0.803-2.219)	0.265		

* Statistically significant correlation. sCEA, preoperative serum CEA; sAFP, preoperative serum CA199; pMMR, Mismatch Repair Proficiency; dMMR, Mismatch Repair Deficiency; Ref, reference variables. Bold values means statistical differences.

**Figure 6:**
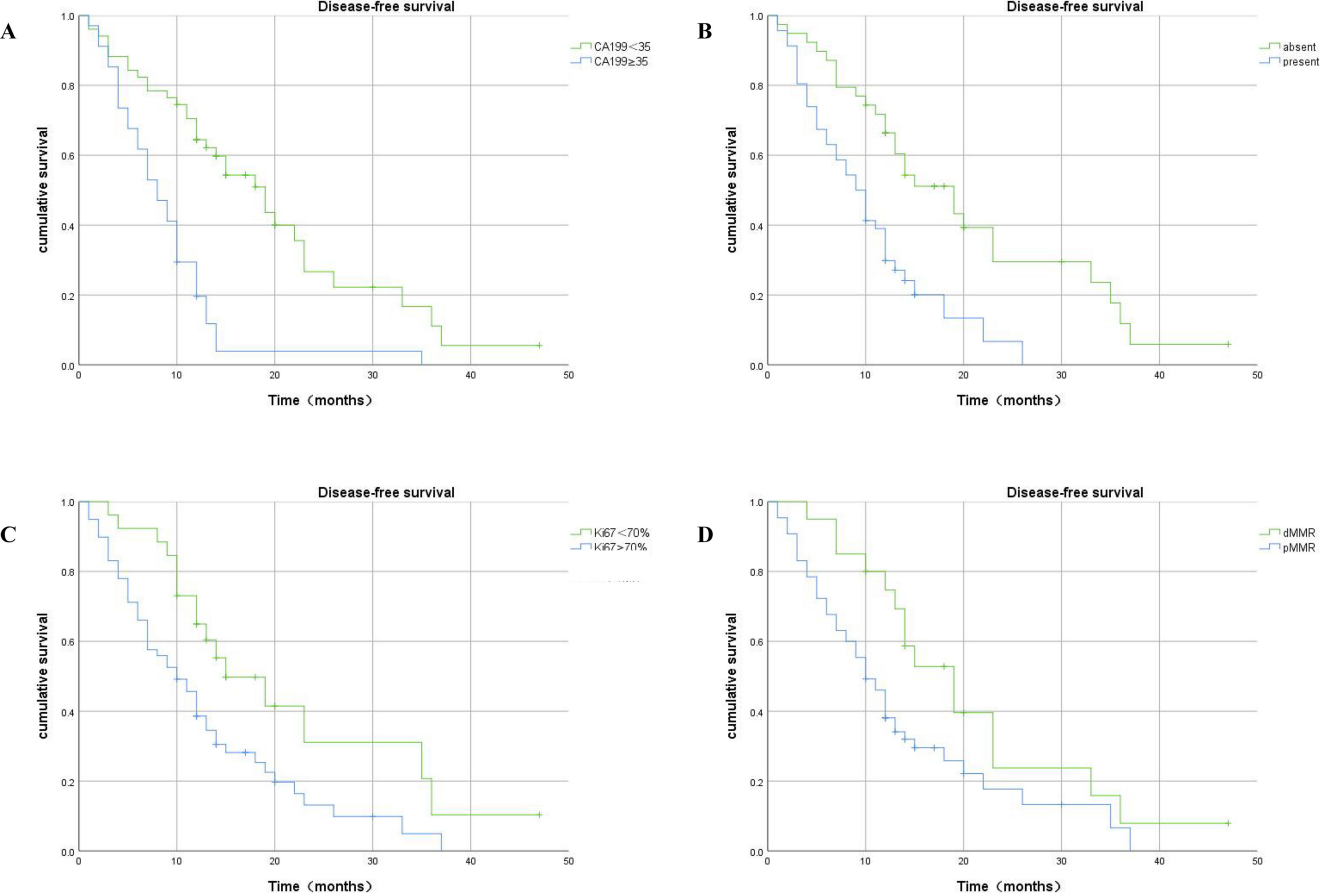
Kaplan–Meier plots of DFS contrasting high versus low expression of CA199, absence versus presence of LVI, high-Ki67 versus low-Ki67, and dMMR versus pMMR. **(A)** Association between DFS and serum CA199 expression: patients with high serum CA199 expression had significantly shorter DFS (p<0.001). **(B)** Association between DFS and LVI expression: patients with present LVI had significantly shorter DFS (p<0.001). **(C)** Association between DFS and Ki67 expression: patients in the high-Ki67 group had shorter DFS (p =0.01). **(D)** Association between DFS and MMR expression: patients with pMMR had significantly shorter DFS (p =0.031).

Kaplan–Meier analysis showed that patients with a high level of serum CA199, high expression of Ki67, LVI, and pMMR had significantly shorter DFS duration than CRLM patients with low serum CA199, absent LVI, low expression of Ki67, and dMMR (**Figure 6**).

### 3.5 Construction of a prognostic nomogram for OS

**Figure 7:**
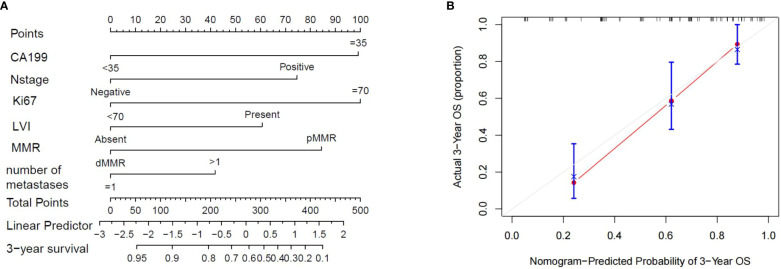
**(A)** The nomogram showing the results of a prognostic model using clinicopathological features to predict the OS of CRLM patients. **(B)**. Calibration curves for 3-year OS based on prognostic models. The X-axis is the model-predicted OS probability, and the Y-axis is the actual OS. The reference lines are marked in gray, and the results are well calibrated.

A prognostic nomogram model was established based on the above independent risk factors for OS (**Figure 7**). Each factor was assigned a score according to the magnitude of its regression coefficient in the Cox model as described below: CA199≥35, 99 points; N1-2 stage, 75 points; Ki67≥70, 100 points; LVI, 61 points; and pMMR, 84 points; more than one liver metastases, 42 points. The total score was obtained by adding the scores of each variable and was then plotted on the total score axis. Subsequently, based on the relationship between the total score and the probability of the outcome events, the predictive value of OS after 3 years for each variable was calculated and plotted on the 3-year survival axis. Importantly, the calibration curve of 3-year OS probability showed a good fit between the nomogram prediction of 3-year OS and our actual observations, implying that the nomogram had a good predictive ability for 3-year OS(C-index:0.732,95CI%0.657-0.792).

**Figure 8:**
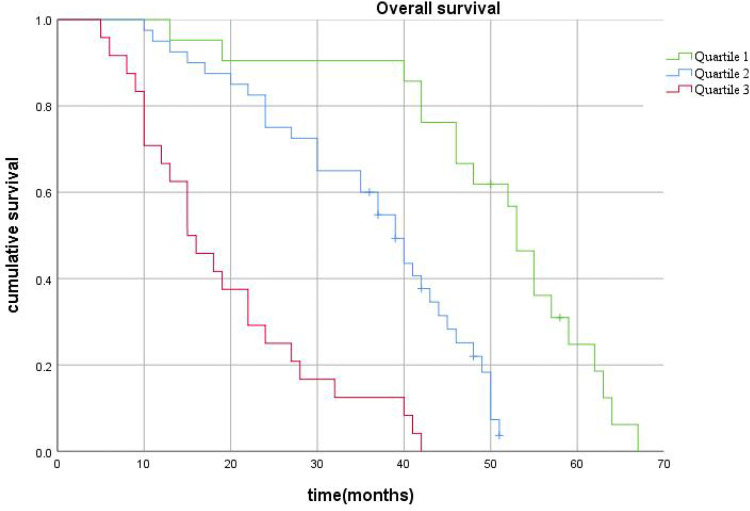
The survival curves stratified by quartiles of the nomogram-predicted score. Patients in Quartile 3 exhibited substantially worse survival than those in Quartile 1 and Quartile 2 (p< 0.001).

After this, patients were stratified according to the quartile of the score predicted by the nomogram. Total scores of 0-201(0-25%) were placed into the Quartile 1 group, 203-358(25%-75%) into the Quartile 2 group, and 361-461(75%-100%) into the Quartile 3 group. Kaplan-Meier curves for OS showed significant differences in survival among the three groups (p<0.001), validating the predictive power of the nomogram model (**Figure 8**).

## 4 Discussion

Until now, the best way to cure colorectal cancer liver metastases has been complete surgical resection. However, the high recurrence rate of patients with CRLM (75-79%) is a major challenge for postoperative treatment with this method and it seriously affects the postoperative prognosis of patients with CRLM (10, 11). Therefore, studying the genes and related expressed proteins in the development of CRLM and identifying the markers or clinicopathological features that may be related to the prognosis of CRLM after surgery are crucial to improving the prognosis of these patients. In this study, multivariate Cox regression analysis showed that in addition to CA199 and N stage, which have been found to be risk factors by many studies, Ki67, LVI and MMR were also independent prognostic factors for OS in patients with CRLM. Furthermore the type of liver metastasis, CA199, Ki67, and LVI were independent prognostic factors for DFS in patients with CRLM. We also developed a nomogram to predict OS in patients with resectable CRLM in this study. In this model, five variables were first identified as independent prognostic factors by multivariate Cox regression analysis, and each prognostic factor had a different predictive effect on patients with resectable CRLM.

MMR is a system that identifies and repairs errors that may occur during DNA replication and recombination, and it plays a crucial part in repairing DNA damage caused by any number of reasons (12, 13). The MMR system consists of a series of specific DNA mismatch repair enzymes, usually dependent on four key genes and corresponding proteins: MLH1, PMS2, MSH2, and MSH6. Although there is no consensus on the use of MMR status as a prognostic factor in patients with CRLM after surgery, studies from Fonzilas suggest that dMMR cannot be used as a prognostic factor to predict the survival of patients with CRC (14). Furthermore, Matteo’s study of microsatellite instability in the tumor tissues of CRLM patients showed that complete loss or partial expression of MMR proteins was not associated with DFS or OS. However, complete or partial expression of MMR protein is more likely to cause intrahepatic recurrence (15). In the genomic analysis of 137 CRLM patients’ tumor tissues by Wang Kun et al. (16), defects in DNA damage repair signaling pathways such as MMR were found to have a significant negative impact on the overall survival of patients. dMMR in particular was found to be a significant indicator for OS in patients with colorectal cancer after surgical treatment in the study of Wen-Yue Yan et al. (17), who found that patients with dMMR also had longer PFS and OS compared to those with pMMR.

In our study, patients with dMMR status had a longer postoperative survival time and a lower risk of recurrence, and this is consistent with some existing results. Previous studies (12, 13) have shown that tumors with dMMR status contain hundreds to thousands of mutations and neoantigens that stimulate the immune system to activate lymphocytes to kill and suppress tumor cells. Therefore, we conclude that MMR status can be used as a prognostic factor for CRLM patients after surgery to guide clinicians in choosing the best treatment and follow-up monitoring strategy to benefit patients.

Another prognostic factor, ki67, is also known as proliferation cell nuclear antigen, and its protein product is localized in the nucleus. It begins to appear in late G1, gradually increases in S and G2 phases, reaches its peak in the M phase, rapidly disappears after the end of mitosis, and is not expressed in the G0 phase (18, 19). Many previous studies have shown that ki67 is overexpressed in many malignant tumors, such as breast cancer, lung cancer, and colorectal cancer (CRC), and that it is related to the development, metastasis, and survival prognosis of tumors (20–22). The level of Ki67 expression is of great significance for evaluating cell proliferation activity, and therefore for studying the biological behavior and prognosis of tumors. Some studies have found that ki67 can provide a reliable proliferation evaluation method for the prognosis of liver metastatic NETs (23). In our study, we identified the best cut-off value of the ki67 proliferation index for predicting postoperative survival in patients with CRLM to be 70%. The survival curve showed that Ki67 was also a strong independent risk factor in CRLM patients, and high-Ki67 was associated with shorter OS and DFS. It may be that tumor cells that overexpress ki67 have a higher ability to divide, which promotes metastasis and results in a worse prognosis (24). It is also possible that tumors that overexpress ki67 are closely related to worse tumor differentiation (25).

Lymphovascular invasion (LVI) refers to the microscopic invasion of blood vessels and lymphatic vessels by tumor cells, which is considered to be closely related to the survival from a variety of malignant tumors (26–28). There is no consensus on the effect of LVI on the postoperative survival of patients with CRLM, however. In a large study from the Memorial Sloan-Kettering Cancer Center (29), multivariate analysis showed that vascular invasion and lymph node invasion of the primary tumor jointly affected the prognosis of CRLM patients. Positive vascular invasion and more lymph node involvement predicted worse prognoses. In another retrospective cohort study from the Chinese Academy of Medical Sciences (30), LVI was independently associated with worse survival as well. Some researchers believe that tumors with LVI have more aggressive behavior and higher recurrence risk, which leads to worse prognoses (31). In this study, we found that the presence of LVI predicted both worse OS and DFS. Patients with LVI were also more likely to relapse after surgery. This may be related to the formation of new formed lymphovascular. Maehara’s study (32) found that there is a high level of VEGF in tumors with LVI, which promotes angiogenesis and that neovascularization with incomplete basement membranes increases the chance of tumor cells invading the vascular system. Other researchers have also suggested that lymphangiogenesis is important in metastatic spread (33). Tumors with LVI may be good candidates for future lymphangiogenesis-related therapies.

In this study, we also found that the results of multivariate analysis showed that serum CA199 was also an independent risk factor affecting the postoperative survival of CRLM patients. The results provide a basis for the prognostic value of preoperative CA199 in CRLM. This is consistent with the results of previous studies. A study involving 691 patients with CRLM showed that when the preoperative CEA level was low, the RFS (P = 0.028) and OS (P = 0.011) of patients with high preoperative CA19-9 level were significantly worse than those with low preoperative CA19-9 level. However, there was no significant difference in RFS (P = 0.758) and OS (P = 0.866) between patients with high preoperative CEA level and patients with high preoperative CA19-9 level. There was no significant difference in prognosis between patients with elevated preoperative CA19-9 or CEA and those with both elevated preoperative CEA and CA19-9. There was also no difference in prognosis between patients with elevated CA19-9 alone and those with elevated CEA alone. This suggests that CA19-9 is a good complement to CEA.

We of course recognize that that our present study suffers from some limitations. First, all clinical data were from a single institution, which may have led to bias. In addition, because the result of the prediction model is based on the factors that have been collected, there is also some room for improvement in the nomogram; there may be other unknown factors that affect the results.

## 5 Conclusion

In this study we performed univariate and multivariate analysis of clinical pathological data and established a corresponding nomogram prediction model whereby we determined that Lymphovascular invasion(LVI), Ki67, and MMR were independent risk factors for the prognosis of patients with CRLM. Furthermore, postoperative recurrence of tumors can be accurately predicted by assessment of Ki67,lvi and MMR. Our findings provide a basis by which to evaluate the prognoses of patients with CRLM and their treatment for related high-risk factors.

## Data availability statement

The original contributions presented in the study are included in the article/**Supplementary material**. Further inquiries can be directed to the corresponding author.

## Ethics statement

The studies involving human participants were reviewed and approved by Medical Ethics Committee of People’s Hospital of Zhengzhou University. Written informed consent for participation was not required for this study in accordance with the national legislation and the institutional requirements.

## Author contributions

DC is the first author. DC and HY conceived and designed the study. DC and QL collected and collated the data. DC performed follow-up and statistical analysis and wrote the manuscript. HY critically revised the manuscript for important intellectual content. All authors contributed to the article and approved the submitted version.

## References

[1] Ferlay J Colombet M Soerjomataram I Parkin DM Piñeros M Znaor A. Cancer statistics for the year 2020: an overview. Int J Cancer (2021). doi: 10.1002/ijc.33588 33818764

[2] Adam R Vinet E. Regional treatment of metastasis: surgery of colorectal liver metastases. Ann Oncol (2004) 15 Suppl 4:iv103–6. doi: 10.1093/annonc/mdh912 15477291

[3] Stewart CL Warner S Ito K Raoof M Wu GX Kessler J. Cytoreduction for colorectal metastases: liver, lung, peritoneum, lymph nodes, bone, brain. when does it palliate, prolong survival, and potentially cure? Curr problems Surg (2018) 55(9):330–79. doi: 10.1067/j.cpsurg.2018.08.004 PMC6422355 30526930

[4] De Jong MC Pulitano C Ribero D Strub J Mentha G Schulick RD. Rates and patterns of recurrence following curative intent surgery for colorectal liver metastasis: an international multi-institutional analysis of 1669 patients. Ann Surg (2009) 250(3):440–8. doi: 10.1097/SLA.0b013e3181b4539b 19730175

[5] Giuliante F Ardito F Vellone M Ranucci G Federico B Giovannini I. Role of the surgeon as a variable in long-term survival after liver resection for colorectal metastases. J Surg Oncol (2009) 100(7):538–45. doi: 10.1002/jso.21393 19722234

[6] Norén A Sandström P Gunnarsdottir K Ardnor B Isaksson B Lindell G. Identification of inequalities in the selection of liver surgery for colorectal liver metastases in Sweden. Scandinavian J Surg SJS (2018) 107(4):294–301. doi: 10.1177/1457496918766706 29692213

[7] Margonis GA Sergentanis TN Ntanasis-Stathopoulos I Andreatos N Tzanninis IG Sasaki K. Impact of surgical margin width on recurrence and overall survival following R0 hepatic resection of colorectal metastases: a systematic review and meta-analysis. Ann Surg (2018) 267(6):1047–55. doi: 10.1097/SLA.0000000000002552 29189379

[8] Chen W Zheng R Baade PD Zhang S Zeng H Bray F. Cancer statistics in China, 2015. CA Cancer J Clin (2016) 66(2):115–32. doi: 10.3322/caac.21338 26808342

[9] Siegel RL Miller KD Goding Sauer A Fedewa SA Butterly LF Anderson JC. Colorectal cancer statistics, 2020. CA Cancer J Clin (2020) 70(3):145–64. doi: 10.3322/caac.21601 32133645

[10] Imai K Allard MA Benitez CC Vibert E Sa Cunha A Cherqui D. Early recurrence after hepatectomy for colorectal liver metastases: what optimal definition and what predictive factors? oncologist (2016) 21(7):887–94. doi: 10.1634/theoncologist.2015-0468 PMC4943389 27125753

[11] Nordlinger B Sorbye H Glimelius B Poston GJ Schlag PM Rougier P. Perioperative chemotherapy with FOLFOX4 and surgery versus surgery alone for resectable liver metastases from colorectal cancer (EORTC intergroup trial 40983): a randomised controlled trial. Lancet (London England) (2008) 371(9617):1007–16. doi: 10.1016/S0140-6736(08)60455-9 PMC2277487 18358928

[12] Dudley JC Lin MT Le DT Eshleman JR. Microsatellite instability as a biomarker for PD-1 blockade. Clin Cancer Res (2016) 22(4):813–20. doi: 10.1158/1078-0432.CCR-15-1678 26880610

[13] Llosa NJ Cruise M Tam A Wicks EC Hechenbleikner EM Taube JM. The vigorous immune microenvironment of microsatellite instable colon cancer is balanced by multiple counter-inhibitory checkpoints. Cancer Discovery (2015) 5(1):43–51. doi: 10.1158/2159-8290.CD-14-0863 25358689 PMC4293246

[14] Fountzilas E Kotoula V Pentheroudakis G Manousou K Polychronidou G Vrettou E. Prognostic implications of mismatch repair deficiency in patients with nonmetastatic colorectal and endometrial cancer. ESMO Open (2019) 4(2):e000474. doi: 10.1136/esmoopen-2018-000474 31231557 PMC6555870

[15] Matteo B Gaetano P Delfina T Riccardo M Roberto S Guglielmo P. Immunohistochemical evaluation of microsatellite instability in resected colorectal liver metastases: a preliminary experience. Med Oncol (2020) 37(7):63. doi: 10.1007/s12032-020-01388-4 32556620

[16] Wang K Liu M Wang HW Jin KM Yan XL Bao Q. Mutated DNA damage repair pathways are prognostic and chemosensitivity markers for resected colorectal cancer liver metastases. Front Oncol (2021) 11:643375. doi: 10.3389/fonc.2021.643375 33869034 PMC8045762

[17] Yan WY Hu J Xie L Cheng L Yang M Li L. Prediction of biological behavior and prognosis of colorectal cancer patients by tumor MSI/MMR in the Chinese population. OncoTargets Ther (2016) 9:7415–24. doi: 10.2147/OTT.S117089 PMC5153316 27994472

[18] Gerdes J Lemke H Baisch H Wacker HH Schwab U Stein H. Cell cycle analysis of a cell proliferation-associated human nuclear antigen defined by the monoclonal antibody ki-67. J Immunol (Baltimore Md 1950) (1984) 133(4):1710–5. doi: 10.4049/jimmunol.133.4.1710 6206131

[19] Miller I Min M Yang C Tian C Gookin S Carter D. Ki67 is a graded rather than a binary marker of proliferation versus quiescence. Cell Rep (2018) 24(5):1105–12.e5. doi: 10.1016/j.celrep.2018.06.110 30067968 PMC6108547

[20] Nielsen TO Leung SCY Rimm DL Dodson A Acs B Badve S. Assessment of Ki67 in breast cancer: updated recommendations from the international Ki67 in breast cancer working group. J Natl Cancer Institute (2021) 113(7):808–19. doi: 10.1093/jnci/djaa201 PMC8487652 33369635

[21] Scagliotti GV Micela M Gubetta L Leonardo E Cappia S Borasio P. Prognostic significance of Ki67 labelling in resected non small cell lung cancer. Eur J Cancer (Oxford Engl 1990) (1993) 29a(3):363–5. doi: 10.1016/0959-8049(93)90387-u 8398336

[22] Melling N Kowitz CM Simon R Bokemeyer C Terracciano L Sauter G. High Ki67 expression is an independent good prognostic marker in colorectal cancer. J Clin Pathol (2016) 69(3):209–14. doi: 10.1136/jclinpath-2015-202985 26281861

[23] Yang Z Tang LH Klimstra DS. Effect of tumor heterogeneity on the assessment of Ki67 labeling index in well-differentiated neuroendocrine tumors metastatic to the liver: implications for prognostic stratification. Am J Surg Pathol (2011) 35(6):853–60. doi: 10.1097/PAS.0b013e31821a0696 21566513

[24] Scopa C D Tsamandas A C Zolota V Kalofonos HP Batistatou A Vagianos C. Potential role of bcl-2 and ki-67 expression and apoptosis in colorectal carcinoma: a clinicopathologic study. Digestive Dis Sci (2003) 48(10):1990–7. doi: 10.1023/a:1026178506348 14627346

[25] Tong G Zhang G Liu J Zheng Z Chen Y Niu P. Cutoff of 25% for Ki67 expression is a good classification tool for prognosis in colorectal cancer in the AJCC−8 stratification. Oncol Rep (2020) 43(4):1187–98. doi: 10.3892/or.2020.7511 PMC7058009 32323802

[26] Mathieu R Lucca I Rouprêt M Briganti A Shariat SF. The prognostic role of lymphovascular invasion in urothelial carcinoma of the bladder. Nat Rev Urol (2016) 13(8):471–9. doi: 10.1038/nrurol.2016.126 27431340

[27] Blakely AM Lafaro KJ Li D Kessler J Chang S Ituarte PHG. Lymphovascular invasion predicts lymph node involvement in small pancreatic neuroendocrine tumors. Neuroendocrinology (2020) 110(5):384–92. doi: 10.1159/000502581 31401633

[28] Skancke M Arnott SM Amdur RL Siegel RS Obias VJ Umapathi BA. Lymphovascular invasion and perineural invasion negatively impact overall survival for stage II adenocarcinoma of the colon. Dis colon rectum (2019) 62(2):181–8. doi: 10.1097/DCR.0000000000001258 30640833

[29] Cardona K Mastrodomenico P D'amico F Shia J Gönen M Weiser MR. Detailed pathologic characteristics of the primary colorectal tumor independently predict outcome after hepatectomy for metastases. Ann Surg Oncol (2013) 20(1):148–54. doi: 10.1245/s10434-012-2540-y 22847127

[30] Mao R Zhao JJ Bi XY Zhang YF Li ZY Zhou JG. Interaction of margin status and tumour burden determines survival after resection of colorectal liver metastases: a retrospective cohort study. Int J Surg (London England) (2018) 53:371–7. doi: 10.1016/j.ijsu.2017.12.001 29229309

[31] Lim SB Yu CS Jang SJ Kim TW Kim JH Kim JC. Prognostic significance of lymphovascular invasion in sporadic colorectal cancer. Dis colon rectum (2010) 53(4):377–84. doi: 10.1007/DCR.0b013e3181cf8ae5 20305435

[32] Maehara Y Kabashima A Koga T Tokunaga E Takeuchi H Kakeji Y. Vascular invasion and potential for tumor angiogenesis and metastasis in gastric carcinoma. Surgery (2000) 128(3):408–16. doi: 10.1067/msy.2000.107265 10965312

[33] Saad RS Kordunsky L Liu YL Denning KL Kandil HA Silverman JF. Lymphatic microvessel density as prognostic marker in colorectal cancer. Modern Pathol (2006) 19(10):1317–23. doi: 10.1038/modpathol.3800651 16799477

