# Supplementary material for: Prognosis of resectable colorectal liver metastases after surgery associated with pathological features of primary tumor
Source: Front Oncol. 2023 May 25;13:1181522. doi: 10.3389/fonc.2023.1181522 (PMC10250016; doi:10.3389/fonc.2023.1181522)
Supplement: Supplementary file 1 [file Table_1.docx]

| Table S1 Comparison of baseline clinicopathologic features between low-Ki67 groups and high-Ki67 groups | | | | | | |
| --- | --- | --- | --- | --- | --- | --- |
| Parameter |  | low-Ki67 groups(n=26) | high-Ki67 groups(n=59) | χ² | p value |  |
| Sex | Female | 10(38.5%) | 20(33.9%) | 0.165 | 0.685 |  |
|  | Male | 16(61.5%) | 39(66.1%) |  |  |  |
| Age(years) | <60 | 16(61.5%) | 29(49.2%) | 1.111 | 0.292 |  |
|  | ≥60 | 10(38.5%) | 30(50.8%) |  |  |  |
| Viral hepatitis | positive | 4(15.4%) | 5(8.5%) | 0.910 | 0.340 |  |
|  | negative | 22(84.6%) | 54(91.5%) |  |  |  |
| Alcohol drinking | no | 17(65.4%) | 40(67.8%) | 0.048 | 0.827 |  |
|  | yes | 9(34.6%) | 19(32.2%) |  |  |  |
| Cigarettes | no | 19(73.1%) | 44(74.6%) | 0.21 | 0.884 |  |
|  | yes | 7(26.9%) | 15(25.4) |  |  |  |
| Timing of liver metastases | Synchronous liver metastases | 18(69.2%) | 35(59.3%) | 0.755 | 0.385 |  |
|  | Metachronous liver metastases | 8(30.8%) | 24(40.7%) |  |  |  |
| Primary lesion site | Left hemi-colon | 23(88.5%) | 40(67.8%) | **4.017** | **0.045** |  |
|  | Right hemi-colon | 3(11.5%) | 19(32.2%) |  |  |  |
| Size of primary tumor (mm) | <50 | 21(80.8%) | 32(54.2%) | **5.412** | **0.020** |  |
|  | ≥50 | 5(19.2%) | 27(45.8%) |  |  |  |
| Size of metastases (mm) | <30 | 14(53.8%) | 34(57.6%) | 0.105 | 0.746 |  |
|  | ≥30 | 12(46.2%) | 25(42.4%) |  |  |  |
| sCEA(ng/ml) | <5 | 3(11.5%) | 15(25.4%) | 2.085 | 0.149 |  |
|  | ≥5 | 23(88.5%) | 44(74.6%) |  |  |  |
| sCA-199(ng/ml) | <35 | 15(57.7%) | 36(61.0%) | 0.083 | 0.773 |  |
|  | ≥35 | 11(42.3%) | 23(39.0%) |  |  |  |
| N stage | N0 | 11(42.3%) | 19(32.2%) | 0.807 | 0.369 |  |
|  | N1-2 | 15(57.7%) | 40(67.8%) |  |  |  |
| T stage | T1-2 | 1(3.8%) | 6(10.2%) | 0.955 | 0.328 |  |
|  | T3-4 | 25(96.2%) | 53(89.8%) |  |  |  |
| Degree of differentiation | High or Moderately differentiation | 25(96.2%) | 52(88.1%) | 1.361 | 0.243 |  |
|  | Poorly differentiation | 1(3.8%) | 7(11.9%) |  |  |  |
| Tumor types | Uplift type | 0(0.0%) | 7(11.9%) | 3.913 | 0.141 |  |
|  | Ulcer type | 24(92.3%) | 50(84.7%) |  |  |  |
|  | invasive | 2(7.7%) | 2(3.4%) |  |  |  |
| Lymphovascular invasion | Absent | 13(50.0%) | 26(44.1%) | 0.256 | 0.613 |  |
|  | Present | 13(50.0%) | 33(55.9%) |  |  |  |
| Nerve invasion | Negative | 10(38.5%) | 24(40.7%) | 0.037 | 0.848 |  |
|  | Positive | 16(61.5%) | 35(59.3%) |  |  |  |
| MMR | dMMR | 12(46.2%) | 8(13.6%) | 10.656 | **0.001** |  |
|  | pMMR | 14(53.8%) | 51(86.4%) |  |  |  |

* Statistically significant correlation. sCEA: preoperative serum CEA; sAFP: preoperative serum CA199; pMMR: Mismatch Repair Proficiency; dMMR: Mismatch Repair Deficiency.
