# Supplementary material for: Prognosis of resectable colorectal liver metastases after surgery associated with pathological features of primary tumor
Source: Front Oncol. 2023 May 25;13:1181522. doi: 10.3389/fonc.2023.1181522 (PMC10250016; doi:10.3389/fonc.2023.1181522)
Supplement: Supplementary file 2 [file Table_2.docx]

| Table S2 Comparison of baseline clinicopathologic features between dMMR groups and pMMR groups | | | | | | |
| --- | --- | --- | --- | --- | --- | --- |
| Parameter |  | dMMR(n=20) | pMMR(n=65) | χ² | p value |  |
| Sex | Female | 9(45.0%) | 21(32.3%) | 1.079 | 0.299 |  |
|  | Male | 11(55.0%) | 44(67.7%) |  |  |  |
| Age(years) | <60 | 14(70.0%) | 31(47.7%) | 3.055 | 0.08 |  |
|  | ≥60 | 6(30.0%) | 34(52.3%) |  |  |  |
| Viral hepatitis | positive | 3(15.0%) | 6(9.2%) | 0.538 | 0.463 |  |
|  | negative | 17(85.0%) | 59(90.8%) |  |  |  |
| Alcohol drinking | no | 15(75.0%) | 42(64.6%) | 0.747 | 0.388 |  |
|  | yes | 5(25.0%) | 23(35.4%) |  |  |  |
| Cigarettes | no | 15(75.0%) | 48(73.8%) | 0.011 | 0.918 |  |
|  | yes | 5(25.0%) | 17(26.2%) |  |  |  |
| Timing of liver metastases | Synchronous liver metastases | 9(45.0%) | 44(67.7%) | 3.355 | 0.067 |  |
|  | Metachronous liver metastases | 11(55.0%) | 21(32.3%) |  |  |  |
| Primary lesion site | Left hemi-colon | 17(85.0%) | 46(70.8%) | 1.615 | 0.204 |  |
|  | Right hemi-colon | 3(15.0%) | 19(29.2%) |  |  |  |
| Size of primary tumor (mm) | <50 | 13(65.0%) | 40(61.5%) | 0.078 | 0.78 |  |
|  | ≥50 | 7(35.0%) | 25(38.5%) |  |  |  |
| Size of metastases (mm) | <30 | 13(65.0%) | 35(53.8%) | 0.774 | 0.379 |  |
|  | ≥30 | 7(35.0%) | 30(46.2%) |  |  |  |
| sCEA(ng/ml) | <5 | 7(35.0%) | 11(16.9%) | 2.994 | 0.084 |  |
|  | ≥5 | 13(65.0%) | 54(83.1%) |  |  |  |
| sCA-199(ng/ml) | <35 | 15(75.0%) | 36(55.4%) | 2.452 | 0.117 |  |
|  | ≥35 | 5(25.0%) | 29(44.6%) |  |  |  |
| N stage | N0 | 12(60.0%) | 18(27.7) | 6.990 | **0.008** |  |
|  | N1-2 | 8(40.0%) | 47(72.3%) |  |  |  |
| T stage | T1-2 | 1(5.0%) | 6(9.2%) | 0.362 | 0.547 |  |
|  | T3-4 | 19(95.0%) | 59(90.8%) |  |  |  |
| Degree of differentiation | High or Moderately differentiation | 19(95.0%) | 58(89.2%) | 0.597 | 0.440 |  |
|  | Poorly differentiation | 1(5.0%) | 7(10.8%) |  |  |  |
| Tumor types | Uplift type | 1(5.0%) | 6(9.2%) | 0.363 | 0.834 |  |
|  | Ulcer type | 18(90%) | 56(86.2%) |  |  |  |
|  | invasive | 1(5.0%) | 3(4.6%) |  |  |  |
| Lymphovascular invasion | Absent | 16(80.0%) | 23(35.5%) | 12.261 | **<0.001** |  |
|  | Present | 4(20.0%) | 42(64.6%) |  |  |  |
| Nerve invasion | Negative | 6(30.0%) | 28(43.1%) | 1.090 | 0.297 |  |
|  | Positive | 14(70.0%) | 37(56.9%) |  |  |  |
| Ki67(%) | <70% | 12(60.0%) | 14(21.5%) | 10.656 | **0.001** |  |
|  | ≥70% | 8(40.0%) | 51(78.5%) |  |  |  |

* Statistically significant correlation. sCEA: preoperative serum CEA; sAFP: preoperative serum CA199; pMMR: Mismatch Repair Proficiency; dMMR: Mismatch Repair Deficiency.
