# Supplementary material for: Prognosis of resectable colorectal liver metastases after surgery associated with pathological features of primary tumor
Source: Front Oncol. 2023 May 25;13:1181522. doi: 10.3389/fonc.2023.1181522 (PMC10250016; doi:10.3389/fonc.2023.1181522)
Supplement: Supplementary file 3 [file Table_3.docx]

| Table S3 Comparison of baseline clinicopathologic features between LVI absent groups and LVI present groups | | | | | | |
| --- | --- | --- | --- | --- | --- | --- |
| Parameter |  | LVI absent groups(n=39) | LVI present groups(n=46) | χ² | p value |  |
| Sex | Female | 16(41.0%) | 14(30.4%) | 1.037 | 0.309 |  |
|  | Male | 23(59.0%) | 32(69.6%) |  |  |  |
| Age(years) | <60 | 23(59.0%) | 22(47.8%) | 1.053 | 0.305 |  |
|  | ≥60 | 16(41.0%) | 24(52.2%) |  |  |  |
| Viral hepatitis | positive | 4(10.3%) | 5(10.9%) | 0.008 | 0.927 |  |
|  | negative | 35(89.7%) | 41(89.1%) |  |  |  |
| Alcohol drinking | no | 27(69.2%) | 30(65.2%) | 0.154 | 0.695 |  |
|  | yes | 12(30.8%) | 16(34.8%) |  |  |  |
| Cigarettes | no | 27(69.2%) | 36(78.3%) | 0.897 | 0.344 |  |
|  | yes | 12(30.8%) | 10(21.7%) |  |  |  |
| Timing of liver metastases | Synchronous liver metastases | 23(59.0%) | 30(65.2%) | 0.350 | 0.554 |  |
|  | Metachronous liver metastases | 16(41.0%) | 16(34.8%) |  |  |  |
| Primary lesion site | Left hemi-colon | 30(76.9%) | 33(71.7%) | 0.296 | 0.587 |  |
|  | Right hemi-colon | 9(23.1%) | 13(28.3%) |  |  |  |
| Size of primary tumor (mm) | <50 | 24(61.5%) | 29(63.0%) | 0.02 | 0.887 |  |
|  | ≥50 | 15(38.5%) | 1737.0%) |  |  |  |
| Size of metastases (mm) | <30 | 22(56.4%) | 26(56.5%) | <0.001 | 0.992 |  |
|  | ≥30 | 17(43.6%) | 20(43.5%) |  |  |  |
| sCEA(ng/ml) | <5 | 8(20.5%) | 10(21.7%) | 0.019 | 0.890 |  |
|  | ≥5 | 31(79.5%) | 36(78.3%) |  |  |  |
| sCA-199(ng/ml) | <35 | 25(64.1%) | 26(56.5%) | 0.505 | 0.477 |  |
|  | ≥35 | 14(35.9%) | 20(43.5%) |  |  |  |
| N stage | N0 | 18(46.2%) | 12(26.1) | 3.721 | 0.054 |  |
|  | N1-2 | 21(53.8%) | 34(73.9%) |  |  |  |
| T stage | T1-2 | 5(12.8%) | 2(4.3%) | 2.005 | 0.157 |  |
|  | T3-4 | 34(87.2%) | 44(95.7%) |  |  |  |
| Degree of differentiation | High or Moderately differentiation | 36(92.3%) | 41(89.1%) | 0.250 | 0.617 |  |
|  | Poorly differentiation | 3(7.7%) | 5(10.9%) |  |  |  |
| Tumor types | Uplift type | 4(10.3%) | 3(6.5%) | 0.434 | 0.805 |  |
|  | Ulcer type | 33(84.6%) | 41(89.1%) |  |  |  |
|  | invasive | 2(5.1%) | 2(4.3%) |  |  |  |
| Nerve invasion | Negative | 22(56.4%) | 12(26.1%) | 8.086 | **0.004** |  |
|  | Positive | 17(70.0%) | 34(56.9%) |  |  |  |
| Ki67(%) | <70% | 13(33.3%) | 13(28.3%) | 0.256 | 0.613 |  |
|  | ≥70% | 26(66.7%) | 33(71.7%) |  |  |  |
| MMR | dMMR | 16(41.0%) | 4(8.7%) | 12.261 | **<0.001** |  |
|  | pMMR | 23(59.0%) | 42(91.3%) |  |  |  |

* Statistically significant correlation. sCEA: preoperative serum CEA; sAFP: preoperative serum CA199; pMMR: Mismatch Repair Proficiency; dMMR: Mismatch Repair Deficiency.
